# Transcriptional responses of *Burkholderia cenocepacia *to polymyxin B in isogenic strains with diverse polymyxin B resistance phenotypes

**DOI:** 10.1186/1471-2164-12-472

**Published:** 2011-09-29

**Authors:** Slade A Loutet, Flaviana Di Lorenzo, Chelsea Clarke, Antonio Molinaro, Miguel A Valvano

**Affiliations:** 1 Centre for Human Immunology, Department of Microbiology and Immunology, the University of Western Ontario, London, Ontario, Canada; 2 Dipartimento di Chimica Organica e Biochimica, Università di Napoli Federico II, Naples, Italy

## Abstract

**Background:**

*Burkholderia cenocepacia *is a Gram-negative opportunistic pathogen displaying high resistance to antimicrobial peptides and polymyxins. We identified mechanisms of resistance by analyzing transcriptional changes to polymyxin B treatment in three isogenic *B. cenocepacia *strains with diverse polymyxin B resistance phenotypes: the polymyxin B-resistant parental strain K56-2, a polymyxin B-sensitive K56-2 mutant strain with heptoseless lipopolysaccharide (LPS) (RSF34), and a derivative of RSF34 (RSF34 4000B) isolated through multiple rounds of selection in polymyxin B that despite having a heptoseless LPS is highly polymyxin B-resistant.

**Results:**

A heptoseless LPS mutant of *B. cenocepacia *was passaged through multiple rounds of selection to regain high levels of polymyxin B-resistance. This process resulted in various phenotypic changes in the isolate that could contribute to polymyxin B resistance and are consistent with LPS-independent changes in the outer membrane. The transcriptional response of three *B. cenocepacia *strains to subinhibitory concentrations of polymyxin B was analyzed using microarray analysis and validated by quantitative Real Time-PCR. There were numerous baseline changes in expression between the three strains in the absence of polymyxin B. In both K56-2 and RSF34, similar transcriptional changes upon treatment with polymyxin B were found and included upregulation of various genes that may be involved in polymyxin B resistance and downregulation of genes required for the synthesis and operation of flagella. This last result was validated phenotypically as both swimming and swarming motility were impaired in the presence of polymyxin B. RSF34 4000B had altered the expression in a larger number of genes upon treatment with polymyxin B than either K56-2 or RSF34, but the relative fold-changes in expression were lower.

**Conclusions:**

It is possible to generate polymyxin B-resistant isolates from polymyxin B-sensitive mutant strains of *B. cenocepacia*, likely due to the multifactorial nature of polymyxin B resistance of this bacterium. Microarray analysis showed that *B. cenocepacia *mounts multiple transcriptional responses following exposure to polymyxin B. Polymyxin B-regulated genes identified in this study may be required for polymyxin B resistance, which must be tested experimentally. Exposure to polymyxin B also decreases expression of flagellar genes resulting in reduced swimming and swarming motility.

## Background

*Burkholderia cenocepacia *belongs to the *B. cepacia *complex (Bcc), a group of Gram-negative opportunistic pathogens infecting patients with cystic fibrosis (CF) and chronic granulomatous disease [1-3]. These infections are detrimental in CF patients because the bacteria can spread between patients via social contact [4], and in some cases patients develop an acute and fatal infection known as "cepacia syndrome" [2]. Treatment of Bcc infections is difficult because the bacteria are resistant to many antibiotics [5-7], including antimicrobial peptides and polymyxins [8-11], a group of compounds that have been proposed as potential new therapeutics for treatment of *Pseudomonas aeruginosa *lung infections in CF patients [12,13].

We have recently proposed a two-tier model of antimicrobial peptide resistance in *B. cenocepacia *[14] with the first and most significant tier consisting of the complete lipopolysaccharide (LPS) core oligosaccharide (OS) [9,15] and the lipid A and core OS aminoarabinose residues that are essential for the viability of *B. cenocepacia *[16,17]. This tier accounts for the low binding of polymyxin B to *B. cenocepacia *cells and poor permeabilization of the *B. cenocepacia *outer membrane [10]. The second tier consists of other mechanisms that each contribute a small amount of antimicrobial peptide resistance but that as whole contribute significantly to the high resistance of this organism [14].

Based on the observation that about 1% of polymyxin B-sensitive *B. cenocepacia *heptoseless LPS mutant cells survive treatment with 500 μg/ml of the antimicrobial peptide polymyxin B for 24 hours (Loutet and Valvano, unpublished), we hypothesized that *B. cenocepacia *heptoseless LPS isolates with increased resistance to polymyxin B could be obtained. We cultured a polymyxin B-sensitive *B. cenocepacia *heptoseless LPS mutant, RSF34 [18], in a way that allowed for the isolation of clones with increased resistance to polymyxin B to identify other mechanisms of antimicrobial peptide resistance in this highly resistant organism. RSF34 has a polymyxin B minimum inhibitory concentration-50 (MIC_50_) of 32 μg/ml which is much lower than the full-length LPS strain from which it was derived, K56-2, which has a polymyxin B MIC_50 _of > 1024 μg/ml [14,18]. *B. cenocepacia *strains with heptoseless LPS make an LPS molecule that consists of lipid A and the innermost core oligosaccharide sugars: a trisaccharide of 3-deoxy-D-*manno*-oct-2-ulopyranosonic acid (Kdo), D-*glycero*-D-*talo*-oct-2-ulopyranosonic acid (Ko) and 4-amino-4-deoxy-L-arabinose (L-Ara4N). Our isolation procedure led to the generation of heptoseless LPS strains with an increasing range of polymyxin B resistance levels, some with at least 40-fold greater resistance than RSF34. Next, we determined how *B. cenocepacia *responds at the transcriptional level after treatment with polymyxin B, as a strategy for identifying additional mechanisms of antimicrobial peptide resistance in *B. cenocepacia*. We used three strains for this study: K56-2, the parental clinical isolate that is highly resistant to polymyxin B [19], RSF34, and RSF34 4000B, the isolate of RSF34 selected for the highest level of polymyxin B resistance. We established the baseline differences in transcription between the strains in the absence of polymyxin B challenge and identified genes transcriptionally regulated by the presence of polymyxin B in the three strains.

## Results

### Isolation and characterization of polymyxin B-resistant *B. cenocepacia *heptoseless LPS clones

Through sequential passage of the heptoseless LPS mutant RSF34, clones were isolated with increasing resistance to polymyxin B. Strain RSF34 25A was isolated from a single colony of RSF34 that grew on LB supplemented with 25 μg/ml polymyxin B. Strain RSF34 200E was isolated from a colony of RSF34 25A that grew on LB with 200 μg/ml polymyxin B, strain RSF34 1000D was isolated as a colony of RSF34 200E that grew on LB containing l000 μg/ml polymyxin B, and strain RSF34 4000B was isolated as a colony of RSF34 1000D that grew on LB containing 4000 μg/ml polymyxin B (Table 1). At each stage of the selection process, an isolate was chosen for the next stage of selection using the following criteria: (1) maintenance of the heptoseless LPS phenotype, (2) a demonstrable increase in polymyxin B resistance using the polymyxin B plate challenge and also, (3) if possible, a demonstrable increase in polymyxin B resistance in the liquid polymyxin B challenge. The number in the nomenclature used for the isolates refers to the concentration of polymyxin B on which the isolate was obtained (in μg/ml); the letter distinguishes different isolates obtained on the same concentration of polymyxin B. To obtain highly polymyxin B-resistant RSF34 isolates required multiple rounds of selection. When RSF34 was plated directly on LB plates with 200 or 1000 μg/ml of polymyxin B no colonies were obtained.

**Table 1:** Strains and plasmids used in this study

Strain or Plasmid	Description	Source or Reference
*Escherichia coli*		
DH5α	F^-^, ϕ80 *lacZ *ΔM15 Δ(*lacZYA-argF*) *U169 deoR endA1 recA1 hsdR17 *(r_K_^- ^m_K_^+^) *thi-1 glnV44*	Laboratory Stock
*Burkholderia cenocepacia*		
K56-2	CF clinical isolate (polymxyin B-resistant)	BCCRC*, [19]
RSF34	K56-2, Δ*hldA *(polymyxin B-sensitive)	[18]
RSF34 25A	RSF34 colony isolated on 25 μg/ml polymyxin B	This study
RSF34 200E	RSF34 25A colony isolated on 200 μg/ml polymyxin B	This study
RSF34 1000D	RSF34 200E colony isolated on 1 mg/ml polymyxin B	This study
RSF34 4000B	RSF34, selected through multiple rounds for polymyxin B resistance	This study
RSF44	K56-2, Δ*fliCD*, flagella-negative	[18]
*Pseudomonas aeruginosa*		
PAO1	Non-CF clinical isolate	[56]
*Plasmids*		
pRK2013	RK2 derivative, Km^R ^*mob*^+ ^*tra*^+ ^*ori*_colE1_	[49]
pSL6	*B. cenocepacia *expression plasmid, rhamnose-inducible promoter, Tp^R ^Cm^R^	[9]
pSL7	pSL6, *B. cenocepacia hldAD*	[9]

*B. cepacia complex Research and Referral Repository for Canadian CF Clinics

**Figure 1:**
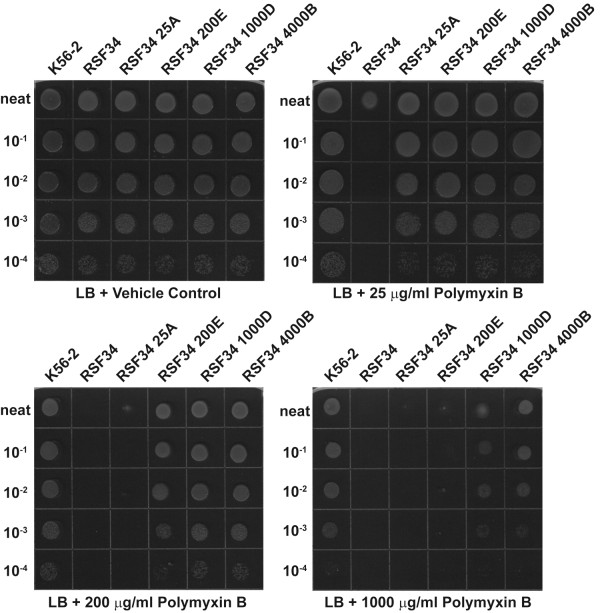
**The resistance of *B. cenocepacia *RSF34 to polymyxin B increases with each round of selection**. Shown is a representative image of three independent experiments, neat is equal to an OD_600 _of 1.0.

The increased resistance after each round of selection is demonstrated in Figure 1. All strains grew equally well on LB plates with a vehicle control (top left). When plated on LB with 25 μg/ml polymyxin B the growth of RSF34 was significantly impaired (top right). The growth of both RSF34 and RSF34 25A was significantly impaired on LB with 200 μg/ml polymyxin B (bottom left). Only K56-2, RSF34 1000D, and RSF34 4000B grew on LB with 1 mg/ml polymyxin B, and RSF34 4000B grew better than RSF34 1000D. No differences were detected in the LPS profiles of any of the resistant isolates (Additional file 1 Figure S1A) and all strains grew similarly under standard laboratory conditions (Additional file 1 Figure S1B). The LPS phenotype could be complemented *in trans *with a plasmid (pSL7) containing both the *hldA *and *hldD *genes (Additional file 1 Figure S1C), while a vector control (pSL6) did not affect the LPS phenotype of any of the resistant isolates (Additional file 1 Figure S1D). Also, RSF34 carrying pSL7 regained polymyxin B resistance to similar levels as the parental strain K56-2 (data not shown), similarly to previously published results with the heptoseless LPS mutant SAL1 [9]. Both *hldA *and *hldD*, which are co-transcribed, encode proteins required for the synthesis of heptose sugars that are incorporated into the LPS core oligosaccharide [9] and the transcription of both genes is defective in RSF34 [18]; therefore, complementation of the LPS phenotype in RSF34 and any strains derived from RSF34 requires that both *hldA *and *hldD *be provided *in trans*. LPS from RSF34 4000B was subjected to a more detailed chemical analysis to determine if there were subtle changes to the LPS molecule that were not detectable on an LPS gel; this LPS did not differ substantially from the typical *B. cenocepacia *lipid A-inner core oligosaccharide [15,20] (Additional file 2 Figure S2 and data not shown). PCR analysis confirmed the presence of the original RSF34 deletion in the polymyxin B-resistant isolates (data not shown).

Next, we tested whether or not the changes are stable or lost in the absence of selective pressure. Cells passaged for five days in the absence of polymyxin B grew as well on plates containing polymyxin B as cells grown only overnight in the absence of polymyxin B (Additional file 3 Figure S3). From this we concluded that the changes that have occurred in our polymyxin B-resistant RSF34 isolates are likely constitutive.

**Figure 2:**
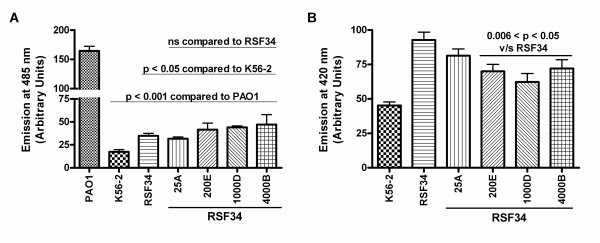
**Dansyl-polymyxin B binding to (A) and 1-N-phenylnaphthylamine permeability of (B) polymyxin B-resistant RSF34 isolates**. (A) Shown are the means and standard error of the means for three experiments, each done in duplicate. P-values for unpaired, Student's t-tests; ns, no significant difference. (B) Shown are the means and standard error of the means for five experiments, each done in duplicate. P-values for unpaired, Student's t-tests.

Binding of polymyxin B was assayed using the fluorescent analogue, dansyl-polymyxin B. When bound to LPS on whole cells, dansyl-polymyxin B will fluoresce at 485 nm after excitation at 340 nm. As previously demonstrated [10,15], we detected high binding of dansyl-polymyxin B to the positive control, *P. aeruginosa *strain PAO1, low binding to *B. cenocepacia *strain K56-2, and a moderate increase in binding to *B. cenocepacia *strain RSF34 (Figure 2A). This increase was similar to that found previously in *B. cenocepacia *mutants with heptoseless LPS [15]. All of the polymyxin B-resistant RSF34 isolates bound dansyl-polymyxin B similarly to RSF34 (Figure 2A), indicating that the levels of dansyl-polymyxin B binding in these mutants are due to the heptoseless LPS and that increased polymyxin B resistance in these mutants is not due to decreased polymyxin B binding.

To measure cell envelope permeability, bacteria were treated with 1-N-phenylnaphthylamine (NPN). In a hydrophobic environment and excited at 350 nm, NPN will emit at 420 nm. When treated with NPN, RSF34 emitted at 420 nm at about twice the level as K56-2 (Figure 2B). The polymyxin B-resistant RSF34 isolates had decreased permeability to NPN compared to RSF34, with the permeability of RSF34 strains 200E, 1000D, and 4000B roughly 30% less than RSF34 (Figure 2B, p-values between 0.006 and 0.05). None of the polymyxin B-resistant isolates had permeability reduced to the level of the wild-type LPS strain K56-2 (Figure 2B). The decreased permeability of the polymyxin B-resistant RSF34 strains, suggest LPS-independent changes in the outer membrane of these mutants leading at least in part to increased polymyxin B resistance.

Consistent with this conclusion, changes in colony morphology were also noted: K56-2 colonies are round with smooth margins (Additional file 4 Figure S4), while those of RSF34 are irregularly shaped, have irregular margins and appear to have the dry and brittle morphology described as "crunchy" by Parker *et al *[21]. The colony morphology of RSF34 25A is similar to RSF34, but RSF34 200E, RSF34 1000D, and RSF34 4000B all form colonies that are more similar to those of K56-2 (Additional file 4 Figure S4). Although the causes of these changes are unknown, they are likely independent of the presence of heptoseless LPS, which is common to all mutant strains.

Together, these results indicate that the selection process provided a series of clonal isolates with: (i) increasing polymyxin B resistance, (ii) a stable polymyxin B resistance phenotype, (iii) no detectable changes in the original heptoseless LPS structure of RSF34, (iv) no significant changes to bacterial fitness, (v) no decreased polymyxin B binding to whole cells, (vi) some decrease in membrane permeability, and (vii) a return to the colony morphology of the parental K56-2 strain. The absence of changes in LPS structure in the RSF34 4000B mutant, suggests additional LPS-independent mechanisms of resistance most likely targeting the bacterial outer membrane.

### RSF34 1000D and RSF34 4000B have increased adherent growth in the presence of high concentrations of polymyxin B

**Figure 3:**
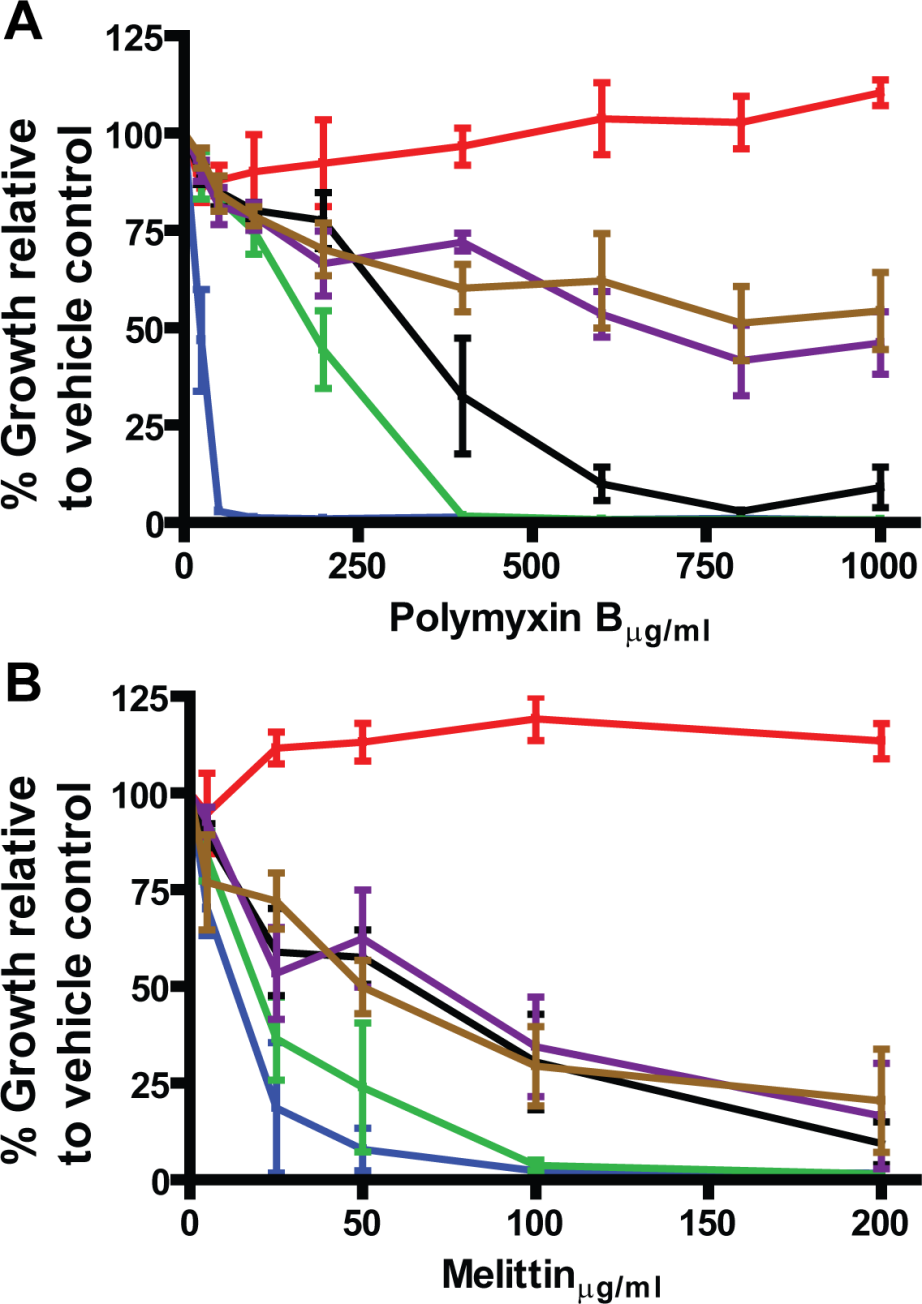
**Polymyxin B-resistant RSF34 isolates have increased polymyxin B resistance in liquid culture and some increase in resistance to melittin**. Growth of K56-2 (red), RSF34 (blue), RSF34 25A (green), RSF34 200E (black), RSF34 1000D (purple), and RSF34 4000B (brown) in liquid culture with between 0 and 1000 μg/ml of polymyxin B (A) or 0 and 200 μg/ml of melittin (B). Data shown are the means for four (polymyxin B) or five (melittin) independent experiments; error bars represent the standard error of the mean.

**Figure 4:**
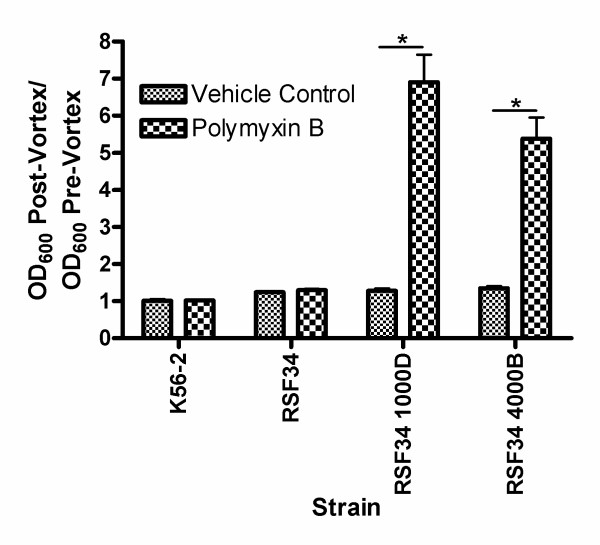
**RSF34 1000D and RSF34 4000B growth in high concentrations of polymyxin B is mainly as microcolonies resuspended in the media or adhered to Eppendorf tube walls**. Cells were grown for 24 hours in either the vehicle control or polymyxin B (25 μg/ml for RSF34 or 600 μg/ml for all other strains). The OD_600 _was measured before vortexing and after vortexing (to resuspend microcolonies and growth that was adherent to tube walls). Shown are the means of the ratios of OD_600 _values post-vortex to pre-vortex for three experiments, with each experiment done in duplicate. Error bars represent the standard errors of the mean. *, Statistically significant difference (p < 0.01) between ratios for growth in the presence of the vehicle control and growth in polymyxin B by unpaired student's t-test.

Similar polymyxin B resistance results were also obtained when the strains were challenged with polymyxin B in liquid culture (Figure 3A). With each round of selection the resistant isolates grew better when challenged in liquid culture up to RSF34 1000D and RSF34 4000B which grew similarly in this assay (Figure 3A). The growth of K56-2 at the highest concentrations of polymxyin B, both in liquid media and on solid media was still considerably better than any of the resistant RSF34 isolates (Figure 1 and 3A). In liquid culture at high concentrations of polymyxin B (≥ 600 μg/ml), significant amounts of the RSF34 1000D and RSF34 4000B growth was either adherent to the sides of tubes or as small microcolonies suspended in the media. Data in Figure 3A represent the optical densities of the media after resuspension of this adherent growth. To quantify this observation, the experiments were repeated for K56-2, RSF34, RSF34 1000D, and RSF34 4000B in the presence of the vehicle control and either 25 μg/ml polymyxin B (RSF34 only) or 600 μg/ml polymyxin B (all other strains). After incubation the OD_600 _was measured from the culture before vortexing tubes and after vortexing tubes. The OD_600 _did not change significantly in K56-2 after vortexing regardless of treatment condition, for RSF34 the OD_600 _increased by about 25% after vortexing, again regardless of treatment condition (Figure 4). This is consistent with the observation of increased cell-to-cell interactions in other Gram-negative bacteria with truncated LPS molecules [22,23]. For RSF34 1000D and RSF34 4000B grown in the presence of the vehicle control there were increases in OD_600 _after vortexing similar to what is seen in RSF34 (Figure 4). However, in the presence of 600 μg/ml polymyxin B, the OD_600 _values after vortexing were 6.9-fold and 5.4-fold greater than before vortexing for RSF34 1000D and RSF34 4000B, respectively (Figure 4).

### Increased resistance is neither specific to polymyxin B nor a general phenomenon

The polymyxin B-resistant isolates were tested for increased resistance to honey bee melittin, an antimicrobial peptide that is structurally unrelated to polymyxin B. There were modest increases in melittin resistance in RSF34 200E, RSF34 1000D, and RSF34 4000B (all of which grew similarly in melittin), but the growth of all of the heptoseless LPS isolates was inhibited by at least 75% in 200 μg/ml melittin (Figure 3B).

Disk diffusion assays were used to test for increased resistance of the polymyxin B-resistant clones to non-peptide antimicrobial compounds and the results are shown in Table 2. No substantial increases in resistance to SDS, novobiocin, tetracycline, or chloramphenicol were seen in any of the polymyxin B-resistant isolates compared to the original heptoseless mutant RSF34. Polymyxin B-resistant isolates RSF34 200E, RSF34 1000D, and RSF34 4000B were all more resistant to imipenem than RSF34 with the latter two strains exhibiting resistance to imipenem similar to the wild-type LPS strain K56-2. Additionally, RSF34 4000B demonstrated increased resistance to gentamicin compared to RSF34 and the other polymyxin B-resistant isolates. Disks spotted with the vehicles in which these antimicrobial compounds were dissolved (water and 95% ethanol) did not impair the growth of any of the strains. Together, these results indicate that the increased resistance to polymyxin B seen in these RSF34 isolates is not completely specific to polymyxin B since they demonstrate some increased resistance to a second antimicrobial peptide as well as some non-peptide antimicrobials, but that the increased resistance is not a general phenomenon since the polymyxin B-resistant isolates are as sensitive as RSF34 to many antimicrobial agents.

**Table 2:** Disk diffusion assay results

Zone of inhibition (mm)*						
Strain	SDS	Novobiocin	Tetracycline	Gentamicin	Chloramphenicol	Imipenem
K56-2	11.3 ± 0.4	22.0 ± 0.4	17.3 ± 1.1	18.6 ± 0.6	17.4 ± 0.3	15.6 ± 0.1
RSF34	21.1 ± 0.3	27.2 ± 0.5	16.8 ± 1.4	20.6 ± 0.1	18.3 ± 0.5	36.7 ± 0.7
RSF34 25A	17.9 ± 0.6	29.3 ± 0.2	17.0 ± 0.7	23.8 ± 0.6	18.2 ± 0.8	35.9 ± 0.5
RSF34 200E	17.8 ± 0.4	30.0 ± 0.4	17.9 ± 0.9	22.7 ± 0.8	19.4 ± 0.1	25.9 ± 1.3
RSF34 1000D	18.3 ± 0.8	30.0 ± 0.4	16.1 ± 1.6	21.2 ± 0.5	19.3 ± 0.5	18.2 ± 0.8
RSF34 4000B	19.9 ± 0.5	29.8 ± 0.3	17.2 ± 1.6	14.2 ± 1.1	20.0 ± 0.5	17.1 ± 0.8

* Values presented are the means and standard error of the means for three or four experiments done in triplicate.

### Experimental approach for microarray analysis

To obtain a comprehensive view of the transcriptional response of *B. cenocepacia *to polymyxin B, and to gain greater insight into the changes that have occurred through our selection process, microarray analysis was conducted on the transcriptional response to treatment with sub-inhibitory concentrations of polymyxin B in three strains (K56-2, RSF34, and RSF34 4000B). For each of the strains we compared transcription in the presence of polymyxin B to transcription in the presence of a vehicle control. Additionally, to obtain baseline differences between the strains we also compared transcription in the presence of the vehicle control between K56-2 and RSF34, and between RSF34 and RSF34 4000B. Concentrations of polymxyin B used in these studies were 25 μg/ml for RSF34 and 500 μg/ml for K56-2 and RSF34 4000B. These were the highest concentrations tested that did not inhibit growth of the strains under the conditions described in the methods (data not shown).

### Establishment of baseline differences between K56-2, RSF34, and RSF34 4000B

**Figure 5:**
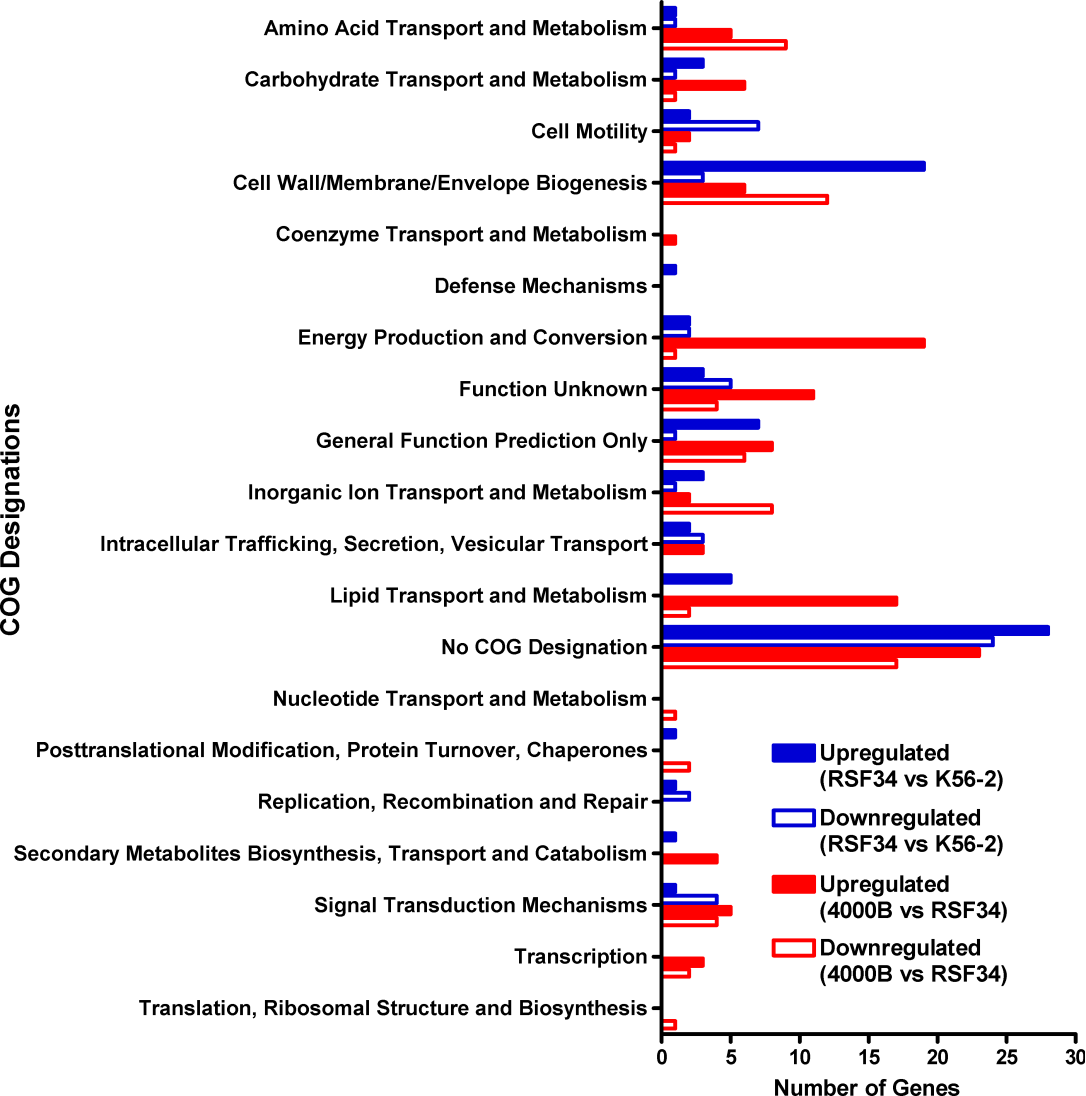
**COG designations of genes differentially regulated under baseline conditions for K56-2, RSF34, and RSF34 4000B**. Genes up- or down-regulated by two-fold or more under vehicle control conditions between RSF34 and K56-2 (blue bars) and RSF34 4000B and RSF34 (red bars) were grouped according to the designations given to their corresponding proteins in the Clusters of Orthologous Groups Database [28] and the *Burkholderia *Genome Database [27].

Before analyzing the transcriptional responses in the presence of polymyxin B in the three strains used in this study, we first investigated the baseline differences in transcription between the strains under the conditions tested in this study. Genes differentially regulated in the presence of the vehicle control between K56-2 and RSF34 are listed in Sheet 1 of Additional file 5 Table S1, while genes differentially regulated in the presence of the vehicle control between RSF34 and RSF34 4000B are listed in Sheet 2 of Additional file 5 Table S1. Genes differentially regulated between the strains were separated into functional classes based on their COG designations (Figure 5). Some interesting patterns of expression were seen when comparing baseline levels of expression in the three strains. Various genes were significantly overexpressed in RSF34 compared to K56-2 (some by over 50-fold) and were also significantly downregulated in RSF34 4000B compared to RSF34 (Additional file 5 Table S1). These include a cluster of genes spanning from *BCAM0529 *to *BCAM0538 *that are predicted to encode proteins with a variety of functions, a cluster of genes spanning from *BCAM0854 *to *BCAM0864 *and known to be involved in biosynthesis of the exopolysaccharide cepacian [24,25], and *BCAM2453 *which is a monocistronic gene that is predicted to encode a protein with redoxin activity. Representative genes from these clusters, *BCAM0537*, *BCAM0855*, and *BCAM2453*, were selected for transcriptional analysis by quantitative Real Time-PCR (qRT-PCR). The patterns of expression for these genes demonstrated by microarray analysis were confirmed by qRT-PCR (Table 3).

**Table 3:** Real-time PCR validation of selected genes identified by microarray analysis as differentially regulated

Gene (predicted function of encoded protein)	Fold-Change	
	**Microarray**^**a**^	**Real-Time PCR**^**b**^
*Genes differentially regulated in RSF34 compared to K56-2*		
		
*BCAL0114 *(Flagellin)	-5.8	No change
*BCAL3490 *(Exported protein)	12.0	6.8
*BCAM0083 *(Hypothetical protein)	12.1	10.0
*BCAM0186 *(Lectin)	14.4	9.8
*BCAM0537 *(Serine peptidase)	51.0	8.0
*BCAM0855 *(UDP-glucose dehydrogenase)	22.2	13.5
*BCAM1010 *(putative UTP-glucose-1-phosphate)	15.2	20.7
*BCAM2453 *(Redoxin)	40.5	6.2
		
*Genes differentially regulated in RSF34 4000B compared to RSF34*		
		
*BCAL1083 *(Exported alkaline phosphatase)	-16.7	-3.2
*BCAL1213 *(2-oxoisovalerate dehydrogenase β subunit)	29.9	19.7
*BCAL1270 *(Phosphate transport, periplasmic)	-23.5	-17.9
*BCAM0537 *(Serine peptidase)	-28.2	-4.1
*BCAM0855 *(UDP-glucose dehydrogenase)	-5.0	-4.2
*BCAM2195 *(AMP-binding enzyme)	22.8	4.9
*BCAM2453 *(Redoxin)	-22.5	-1.9
		
*Genes differentially regulated in K56-2 upon polymyxin B treatment*		
		
*BCAL0114 *(Flagellin, FliC)	-4.9	-10.3
*BCAL0140 *(Flagellar biosynthesis)	-25.9	-26.2
*BCAL0520 *(Flagellar hook-length control, FlhB)	-15.1	-131.9
*BCAL0566 *(Flagellar basal body rod modification, FlgD)	-17.1	-7.7
*BCAL1351 *(Exported Protein)	3.6	31.3
*BCAL3507 *(FliL)	-13.6	-4.3
*BCAM0083 *(Hypothetical protein)	21.4	34.5
*BCAM2187 *(Macrolide-specific ABC-type efflux)	9.5	33.7

^a^Data shown are the mean from three independent experiments.

^b^Data shown are the mean of two or three independent experiments, which are also independent of those used for microarray analysis.

Other genes significantly overexpressed in RSF34 compared to K56-2 that are not then altered between RSF34 4000B and RSF34 include many genes predicted to encode exported proteins, lipoproteins, as well as proteins involved in cell envelope biogenesis, carbohydrate transport and metabolism, an efflux system, and a lectin (Additional file 5 Table S1). *BCAL3490*, *BCAM0083*, *BCAM0186*, and *BCAM1010 *were chosen as representative up-regulated genes and this was confirmed by qRT-PCR (Table 3).

There were few genes downregulated to a large extent in RSF34 compared to K56-2 (Additional file 5 Table S1). The gene *BCAL2945 *shown in Additional file 5 Table S1 to be down-regulated 87.4-fold in RSF34 compared to K56-2 is *hldA*, the gene deleted in RSF34. Otherwise the only other gene down-regulated by 5-fold or more in RSF34 is *BCAL0114 *that encodes flagellin. This is consistent with the observation that RSF34 is less motile than K56-2 (Additional file 6 Figure S5); however, qRT-PCR found no differences in *BCAL0114 *expression between RSF34 and K56-2 (Table 3). All other genes are downregulated by 4-fold or less and more than 50% of these genes were predicted prophage-related genes in the genomic island BcenGI12, which spans from *BCAM1024 *to *BCAM1096 *[26,27]. Again, there is reciprocal regulation of the genes in BcenGI12 and they are upregulated in RSF34 4000B compared to RSF34 (Additional file 5 Table S1).

Finally there were genes differentially expressed in RSF34 4000B compared to RSF34 that were not significantly altered between RSF34 and K56-2 (Additional file 5 Table S1). These genes include two clusters (*BCAL1212 *to *BCAL1215 *and *BCAM2191 *to *BCAM2196*) that are overexpressed by 10-fold or more and are predicted to encode proteins involved in energy production and lipid metabolism. Genes down-regulated by 10-fold or more include one cluster (*BCAL1270 *to *BCAL1276*) predicted to encode a phosphate ABC transport system and *BCAL1083*, which encodes a predicted exported alkaline phosphatase. qRT-PCR analysis of representative genes from these clusters (*BCAL1213*, *BCAL2195*, *BCAL1270*, and *BCAL1083*) confirmed these patterns of expression (Table 3). These genes are of interest because their changes in expression could contribute to the increased resistance of RSF34 4000B to polymyxin B compared to RSF34. With the baseline differences between K56-2, RSF34, and RSF34 4000B established, we next sought to investigate the polymyxin B transcriptional responses made by each of these three strains.

### Cell motility associated genes are downregulated by *B. cenocepacia *K56-2 upon polymyxin B treatment

**Figure 6:**
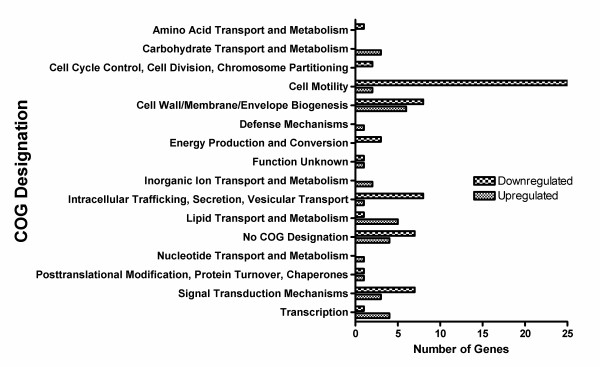
**COG designations of genes differentially regulated upon treatment with polymxyin B in K56-2**. Genes up- or down-regulated by two-fold or more upon treatment with polymyxin B compared to treatment with the vehicle control were grouped according to the designations given to their corresponding proteins in the Clusters of Orthologous Groups Database [28] and the *Burkholderia *Genome Database [27].

*B. cenocepacia *K56-2 was grown to mid-log phase, treated with 500 μg/ml of polymyxin B or a vehicle control, RNA was extracted, converted to cDNA, and subjected to microarray analysis. For a complete list of genes differentially regulated in the presence of polymyxin B compared to the vehicle control in K56-2 see Sheet 1 of Additional file 7 Table S2. The differentially regulated genes were organized according to their Clusters of Orthologous Groups (COG) designations (Figure 6) [28]. The largest group of differentially regulated genes was those associated with cell motility. Almost all genes encoding proteins required for building and operating flagella are divided into five clusters located on the largest chromosome of *B. cenocepacia *(*BCAL0113*-*BCAL0114*, *BCAL0140*-*BCAL0144*, *BCAL0520*-*BCAL0527*, *BCAL0561*-*BCAL0577*, *BCAL3501*-*BCAL3507*) [26]. Genes from all five of these clusters were downregulated upon treatment with polymxyin B (Additional file 7 Table S2). Furthermore, the only genes differentially downregulated by 10-fold or more in the presence of polymyxin B were genes in these clusters. Downregulation was confirmed by qRT-PCR (Table 3) for one gene from each of the five clusters (*BCAL0114*, *BCAL0140*, *BCAL0520*, *BCAL0566*, and *BCAL3507*). Based on these results we concluded that under the conditions we tested, one of the major transcriptional responses of *B. cenocepacia *to polymyxin B was a downregulation of cell motility genes, particularly those required for flagella. Two assays were conducted to test for a role for flagella in polymyxin B resistance. First, MIC_50 _values were determined for *B. cenocepacia *strain K56-2 and an isogenic, non-flagellated mutant, RSF44 [18]. Both strains had MIC_50 _values greater than 1024 μg/ml for polymyxin B, which was the highest concentration tested. Second, bacteria were grown overnight in liquid culture and then serial dilutions were plated on solid agar plates containing either 500 μg/ml polymyxin B or a vehicle control. Both strains grew equally well under either of the conditions (data not shown). Together, these experiments suggested that a mutant that lacks flagella maintains polymyxin B resistance at least at the concentrations tested and that the concentrations of polymyxin B tested in the following assays do not significantly inhibit the growth of either of the strains.

### Swimming and swarming motility are impaired by polymyxin B

**Figure 7:**
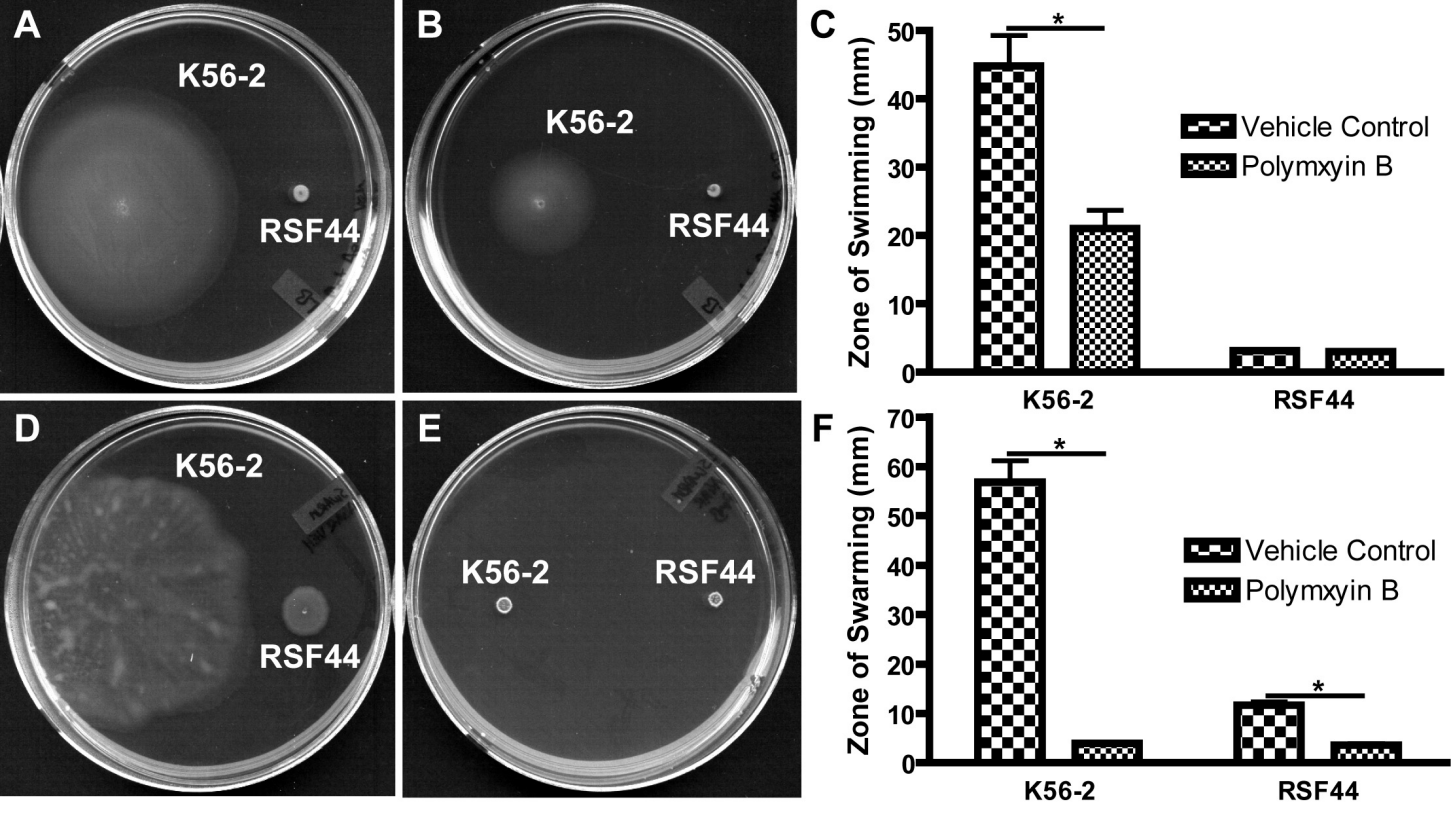
**Swimming and swarming motility are inhibited in the presence of polymyxin B**. Bacteria were inoculated into swimming motility plates (A and B) or on swarming motility plates (D and E) containing either the vehicle control (A and D) or 500 μg/ml polymyxin B (B and E). Images shown are representatives from six independent experiments. Diameters across the zones of bacterial swimming or swarming were measured and the means and standard errors of the means for all six independent experiments are shown in (C and F). *Statistically significant difference, p < 0.01 by student's t-test between vehicle control and polymyxin B treatments.

Of the five types of bacterial motility that have been described (swimming, swarming, twitching, gliding, and sliding) [29], two, swimming and swarming, are dependent on flagella [30] and have been demonstrated to occur in *B. cenocepacia *[31,32]. We tested the effect of polymyxin B on the ability of *B. cenocepacia *to move by both of these mechanisms. The parental strain K56-2 and a flagella-negative mutant, RSF44, were inoculated into semi-solid agar plates containing either a vehicle control or 500 μg/ml of polymyxin B throughout the media to test for swimming motility. Growth of K56-2 in plates with the vehicle control radiated outwards from the point of inoculation to a much greater extent than it did in plates containing 500 μg/ml polymyxin B (Figure 7A and 7B), while RSF44 grew as a single colony at the point of inoculation regardless of treatment condition (Figure 7A and 7B). Quantitation of this phenotype (Figure 7C) showed that on average the zone of growth of K56-2 in swimming motility plates with the vehicle control was more than twice the size of the growth in plates with polymyxin B.

Assays were also conducted to determine if swarming motility is impaired by polymyxin B. K56-2 was able to swarm across plates containing the vehicle control (Figure 7D and 7F). The flagella-negative mutant RSF44 also grew across the surface of the plates with the vehicle control, although to a much less extent than K56-2 (Figure 7D and 7F). Motility of both of these strains was highly impaired in the presence of 500 μg/ml polymyxin B (Figure 7E and 7F). Swarming motility results in the absence of polymyxin B for RSF44 are similar to those reported for a *Pseudomonas aeruginosa *mutant lacking both flagella and the type IV pili, which began to undergo sliding motility when grown under conditions required for swarming motility [33]. The authors of this study also found that sliding and swarming motility resulted from similar environmental cues, which could explain why both K56-2 and RSF44 appear non-motile in the presence of polymyxin B. These types of analyses were not completed for RSF34 and RSF34 4000B because both strains have motility similar to RSF44 in the absence of polymyxin B (Additional file 6 Figure S5).

### Polymyxin B treatment upregulates genes with diverse functions

Treatment of K56-2 with polymyxin B led to the upregulation of thirty genes (Sheet 1 of Additional file 7 Table S2), predicted to encode proteins involved in a variety of pathways, particularly lipid transport and metabolism and cell envelope biogenesis (Figure 6). Genes that were amongst the most highly overexpressed include: a cluster spanning *BCAM0082 *to *BCAM0084 *that contains genes encoding proteins with predicted sugar modifying and transferase activities, *BCAM1364*, encoding a predicted NAD dependent epimerase/dehydratase, and *BCAM2186 *to *BCAM2188*, encoding a predicted macrolide efflux system. qRT-PCR conducted for *BCAM0083 *and *BCAM2187 *demonstrated that both genes were overexpressed (Table 3). qRT-PCR experiments were also attempted for *BCAM1364*. However, these experiments failed, as we could not obtain a primer pair that efficiently amplified the transcript or the gene from genomic DNA (data not shown). The genes upregulated upon treatment with polymyxin B are of interest as they may represent novel genes involved in the resistance of *B. cenocepacia *to polymyxin B.

### Regulation by polymyxin B in RSF34 and RSF34 4000B

Since we have previously identified genes involved in polymyxin B resistance in RSF34 [14], we thought that it might be possible to identify additional polymyxin B-responsive genes in this strain that were not identified in K56-2. In total, 59 genes were upregulated in RSF34 upon polymyxin B treatment (Sheet 2 of Additional file 7 Table S2) and of the 30 genes upregulated by K56-2 in the presence of polymyxin B, more than a third appeared in the RSF34 data set as well. Of the remaining 48 genes upregulated in RSF34 in the presence of polymyxin B only two are upregulated by five-fold or more. The only cluster that stood out in this data set was a group of genes, *BCAL1349 *to *BCAL1351*, predicted to encode a two-component regulatory system and an outer membrane protein, transcribed in opposite orientations. This cluster is also upregulated in K56-2 in the presence of polymyxin B but not to the same extent (less than four-fold). qRT-PCR experiments for *BCAL1351 *using RNA extracted from K56-2 grown in the presence or absence of polymyxin B indicated that this cluster of genes is upregulated in the presence of polymyxin B (Table 3). Almost all genes downregulated by RSF34 in the presence of polymyxin B are involved in the assembly or function of the flagellum (Sheet 2 of Additional file 7 Table S2).

The picture is quite different in RSF34 4000B; 204 genes were found to be upregulated in the presence of polymyxin B, all of which were upregulated by less than five-fold (Sheet 3 of Additional file 7 Table S2). This list of genes did not include some of the largest changes described above, including the cluster spanning *BCAM0082 *to *BCAM0084*, or the fold-changes were less than those seen in K56-2 and/or RSF34. Only 32 genes were downregulated by RSF34 4000B in the presence of polymyxin B, a third of which were tRNA-encoding genes, and none of which were flagellar-related genes (Sheet 3 of Additional file 7 Table S2).

## Discussion

Two genomic approaches were utilized to study polymyxin B resistance in *B. cenocepacia*. A lineage of increasingly polymyxin B-resistant heptoseless LPS mutants was obtained through selection of resistant isolates on media that prohibited the replication of the vast majority of the cells plated. At each round of selection approximately one polymyxin B-resistant isolate per 10^5 ^CFU plated was obtained. Initial characterization of these isolates shows that they exhibited: (i) increased polymyxin B resistance that is relatively stable, since it is maintained after the cells are grown in the absence of polymyxin B for five days; (ii) no defects in growth rate, suggesting that the mutation or mutations do not affect the general fitness of the bacteria, and (iii) increased polymyxin B resistance in liquid media despite the fact that selection of the isolates occurred on solid media. These isolates show significant increases in resistance to imipenem and melittin, and in the case of the final isolate (RSF34 4000B), to gentamicin.

Although it must be noted that the pleiotropic changes seen in the polymyxin B-resistant isolates may not all contribute to the polymxyin B resistance of the isolates, many of the phenotypic changes seen in the isolates could be associated with increased antimicrobial peptide resistance. Subpopulations of *P. aeruginosa *and *E. coli *within biofilms [34,35] have been shown to develop increased antimicrobial peptide resistance and the results presented here suggest that high concentrations of polymyxin B induce increased adherent growth of RSF34 1000D and RSF34 4000B, possibly resulting in protection of bacterial cells within the adherent growth from polymyxin B. The heptoseless LPS phenotype induces outer membrane instability that has pleiotropic effects on bacteria [36,37] including changes in colony morphology changes [21,38,39]. The return to the wild-type colony morphology in RSF34 200E and later isolates could indicate that the polymyxin B-resistant isolates may have altered outer membrane properties that in some way stabilize the outer membrane. This interpretation is consistent with our observation of decreased NPN access to the outer membrane in the polymyxin B-resistant isolates. Experiments are currently underway to precisely determine the genes and/or proteins whose expression and/or function have been altered through the selection process to increase the polymyxin B resistance of RSF34.

Microarray analysis conducted to compare baseline changes in the gene expression between K56-2, RSF34 and RSF34 4000B show that there are many genes whose expression are substantially upregulated in RSF34 compared to K56-2 (Additional file 5 Table S1, and Table 3). The types of genes differentially expressed in RSF34 compared to K56-2 are consistent with observations in the literature that heptoseless LPS mutants tend to alter the synthesis of other polysaccharides [21,38,39], The data are also similar to a microarray study of a *Salmonella enterica *serovar Typhimurium heptoseless LPS mutant which showed changes in sugar metabolism and expression of genes predicted to encode outer membrane proteins and lipoproteins, as well as decreased expression of genes required for the flagellum [40]. These types of changes are likely the consequence of the pleiotropic effects seen in heptoseless LPS mutants [36,37]. Interestingly, RSF34 4000B reverses some but not all of these changes (Additional file 5 Table S1, and Table 3). This is similar to some of the phenotypic changes seen in this strain such as return to wild-type colony morphology (Additional file 4 Figure S4) and intermediate permeability to NPN (Figure 2B). Since some of the genes overexpressed in RSF34 are downregulated in RSF34 4000B, this isolate could have an altered cell envelope that decreases these pleiotropic effects, and also makes it less susceptible to polymyxin B challenge. There are also genes (*BCAL1212 *to *BCAL1215*, *BCAM2191 *to *BCAM2196*, *BCAL1270 *to *BCAL1276*, and *BCAL1083*) whose expression is significantly altered in RSF34 4000B compared to RSF34 but are not altered between K56-2 and RSF34. These changes in gene expression may contribute to the increased polymyxin B resistance in RSF34 4000B, which we are currently studying using mutagenesis strategies.

Downregulation of motility-associated gene expression is a major transcriptional response in *B. cenocepacia *upon treatment with polymyxin B under the conditions tested in this study which results in impairment of swimming and swarming motility in the presence of polymyxin B. None of the genes that have previously been implicated in the resistance of *B. cenocepacia *to antimicrobial peptides [9,14-16,41] were differentially regulated by polymyxin B under the conditions tested. It is possible that other conditions (such as higher concentrations of polymyxin B or treatment with other antimicrobial peptides) are required to see differential regulation of these genes. It is also just as possible that expression of these antimicrobial peptide resistance genes are not regulated by the presence of antimicrobial peptides in the environment and that major resistance mechanisms are constitutively active in *B. cenocepacia*, which could help to explain in part the high resistance of *B. cenocepacia *to these compounds. There are various genes upregulated by both K56-2 and RSF34 grown in the presence of polymyxin B, including genes encoding proteins involved in lipid transport and metabolism, cell envelope biogenesis, signal transduction, and transcription, as well as genes of unknown function. Characterization of potential roles in polymyxin B resistance for these genes is currently underway.

At least two microarray studies have been published on *P. aeruginosa *and its response to antimicrobial peptides that have identified differential regulation of genes associated with motility. Cummins *et al *showed that exposure of planktonic cells to subinhibitory concentrations of colistin (polymyxin E) lead to small decreases in expression of genes associated with motility [42]. The authors of this study also found that the response of *P. aeruginosa *to colistin included upregulation of both known colistin resistance genes as well as genes that had not previously been shown to be involved in resistance (such as genes involved in quorum sensing and biofilm formation). Meanwhile, Overhage *et al *showed that exposure of *P. aeruginosa *grown as biofilms in the presence of subinhibitory concentrations of LL-37 led to increased expression of type IV pili genes and decreased expression of flagella genes [43]. Phenotypically, the presence of subinhibitory concentrations of LL-37 led to increased twitching motility (which requires pili [30]), but had no effect on swarming or swimming motility.

Swarming motility is a multicellular bacterial lifestyle [30] and there are conflicting reports in the literature as to whether or not this protects bacteria from antimicrobial peptides. Lai and colleagues [44] showed that swarming cells of *Escherichia coli*, *P. aeruginosa*, and *Bacillus subtilis *were more resistant than planktonic cells to numerous antibiotics, except for the antimicrobial peptides polymyxin B and colistin; while Kim *et al *[45] showed that swarming *Salmonella enterica *serovar Typhimurium cells were more resistant than planktonic cells to polymxyin B and colistin. Our data indicates that a subinhibitory concentration of polymyxin B greatly impairs the ability of *B. cenocepacia *to both swim and swarm.

## Conclusions

We demonstrate that it is possible to obtain heptoseless LPS strains of *B. cenocepacia *with high resistance to polymyxin B, and suggest that this may occur through LPS-independent changes that stabilize the outer membrane in some way. Furthermore, our data demonstrate that major transcriptional changes made by *B. cenocepacia *upon treatment with polymxyin B include downregulation of genes required for the synthesis and operation of the flagella and upregulation of a set of genes encoding proteins with diverse predicted functions. The contribution made by genes that are upregulated by *B. cenocepacia *upon treatment with polymyxin B to polymyxin B resistance must now be determined and is underway in the Valvano Laboratory. Decreased flagellar gene expression upon treatment with polymyxin B impairs both swimming and swarming motility, two processes that require the flagella. *B. cenocepacia *mutants lacking flagella have been shown to be less virulent in mice [46] and less able to invade A549 human respiratory epithelial cells [47]. Additionally, upregulation of flagellar genes has been reported in *B. cenocepacia *when it is grown in CF sputum [48]. Therefore, even if a therapeutically available antimicrobial peptide was incapable of killing *B. cenocepacia *it might still be useful for treating *B. cenocepacia *infections because the inhibition of motility may be detrimental to the pathogenicity of *B. cenocepacia*.

## Methods

### Bacterial strains, culture conditions, and reagents

All strains and plasmids used in this study are listed in Table 1. A clinical isolate of *B. cenocepacia*, strain K56-2 [19], and strains derived from K56-2, were used for microarray analysis. K56-2 is clonally related to the sequenced *B. cenocepacia *strain J2315 [26] whose sequence was used to design the *B. cenocepacia *microarrays [48]. Unless otherwise noted, all bacterial cell culturing was done at 37°C in either Luria Broth (LB) or LB solidified with 1.6% Bacto Agar. When required, antibiotics were used at the following concentrations: trimethoprim, 100 μg/ml for *B. cenocepacia *and 50 μg/ml for *E. coli*; kanamycin, 40 μg/ml for *E. coli*. All antibiotics and chemicals were obtained from Sigma-Aldrich (St. Louis, Missouri, USA). All media was purchased from Becton, Dickinson, and Company (Franklin Lakes, New Jersey, USA). Polymyxin B was dissolved in 0.2% bovine serum albumin + 0.01% acetic acid, which was also used as a vehicle control in all experiments.

### Isolation of colonies with increased resistance to polymyxin B

Four sequential rounds of selection were used to obtain RSF34 isolates able to grow on up to 4 mg/ml of polymyxin B. For each round of selection, cells were grown overnight to stationary phase and then diluted to approximately 5 × 10^6 ^CFU/mL in LB and 100 μl of cells (approximately 5 × 10^5 ^CFU) was plated on an LB agar plate supplemented with polymyxin B. The plate was incubated for 40 to 48 h and the colonies (typically 4-6 colonies) were selected for further study. In the first round of selection, RSF34 cells were plated on 25 μg/ml polymyxin B. In the second round of selection, one of the RSF34 isolates from the first round of selection, RSF34 25A, was used and bacteria were plated on 200 μg/ml polymyxin B. Next, an isolate from the second round of selection (RSF34 200E) was plated on 1000 μg/ml polymyxin B. Finally, an isolate from the third round of selection (RSF34 1000D) was plated on 4000 μg/ml polymyxin B and isolate RSF34 4000B was selected for study.

For complementation studies, plasmids pSL6 and pSL7 were transferred to RSF34 and the RSF34 resistant isolates by triparental mating with the pRK2013 helper plasmid [49]. Gentamicin (50 μg/ml) was used to select against the *E. coli *donor and helper strains. Rhamnose (0.2% wt/vol) was used to induce gene expression from the plasmids.

### LPS analysis

For analysis by gel electrophoresis and silver staining, LPS was prepared and visualized as previously described [9,50]. For more detailed chemical analyses, monosaccharides and fatty acids were identified using gas-liquid chromatography-mass spectrometry (GLC-MS) and matrix-assisted laser desorption/ionization-time of flight-mass spectrometry (MALDI-TOF-MS) as previously described [15,20].

### PCR analysis of RSF34 mutation

Maintenance of the RSF34 mutation in the polymyxin B-resistant isolates was confirmed by PCR amplification with primers 1689 and 1690 (Table 4) using Taq Polymerase (QIAGEN Inc., Mississauga, Ontario, Canada) and the following thermal cycling conditions: 94°C for 2 min, 29 cycles of 94°C for 40 sec, 60°C for 40 sec, and 72°C for 90 sec, followed by a final extension at 72°C for 7 min. These primers bind outside the region to be deleted, giving amplification in the wild-type strain of a band of approximately 1500 base pairs and in RSF34 and its derivatives, a band of approximately 600 base pairs.

**Table 4:** Primers used in this study

Primer	Sequence
1689	5'-GCGCGTACCTTGCCGAAATC-3'
1690	5'-CTACGATCCGGTCGCAGTCG-3'
*hisD *for	5'-AGCTGGCAGTACACGGAAAG-3'
*hisD *rev	5'-GCACGACCATCACGATCTC-3'
*BCAL0114 *for	5'-GTTGCACAGCAGAACCTCAA-3'
*BCAL0114 *rev	5'-AGACCGTTGATCTGGGTCTG-3'
*BCAL0140 *for	5'-ACGTGCCTTACCAACTCTGG-3'
*BCAL0140 *rev	5'-CATCTCGCCATCCGTGTATT-3'
*BCAL0520 *for	5'-ACTGGACGGATGCACTAAGC-3'
*BCAL0520 *rev	5'-GTGCTGCGACACGAACAG-3'
*BCAL0566 *for	5'-AACACCAACAACGTGTCGTC-3'
*BCAL0566 *rev	5'-GTGAGCGACGTGTTCAACTG-3'
*BCAL1083 *for	5'-GCCAGTTCTATTCCGACTGC-3'
*BCAL1083 *rev	5'-CGTGTCGACGTTGTGGTACT-3'
*BCAL1213 *for	5'-TACCGAAGGACTGCAGAACA-3'
*BCAL1213 *rev	5'-TCGGATGCCGGATAGAAATA-3'
*BCAL1270 *for	5'-ACCAGATCCTGACGAACCAG-3'
*BCAL1270 *rev	5'-CGTCACCTTCGTCTTCCACT-3'
*BCAL1351 *for	5'-AACGGCTTCTTCATCGACAG-3'
*BCAL1351 *rev	5'-CCCATCCCCTTCAGGTAGTC-3'
*BCAL3490 *for	5'-GGTGCAGTTCTCGGTGTAGC-3'
*BCAL3490 *rev	5'-ACTCGTGTTCACGCCACTG-3'
*BCAL3507 *for	5'-GAACAAGCATCCCGAGGAG-3'
*BCAL3507 *rev	5'-ACGAACTCGGTGAACAGGAC-3'
*BCAM0083 *for	5'-ATCCGCATCTATCACTTCGG-3'
*BCAM0083 *rev	5'-TACGCGAGGTAGGTCTTGCT-3'
*BCAM0186 *for	5'-TGGCTGATTCTCAAACGTCA-3'
*BCAM0186 *rev	5'-ACACCTCGAAACGGATCTTG-3'
*BCAM0537 *for	5'-GCGTGATTCCGCTGCTGGA-3'
*BCAM0537 *rev	5'-GTTGCCCGCGTCGCTGAT-3'
*BCAM0855 *for	5'-ACCAGATGTTCTCGGTCGTGTCG-3'
*BCAM0855 *rev	5'-ATTCGCCGCGTACTTCGTGAA-3'
*BCAM1010 *for	5'-GAAAAGCTGCTCGAACTCGT-3'
*BCAM1010 *rev	5'-TCACCGAGCTGTGATAGTGG-3'
*BCAM2187 *for	5'-GTCGTCCTGAACAACGTCAA-3'
*BCAM2187 *rev	5'-GCAAGTGATAGCGCTGGAAT-3'
*BCAM2195 *for	5'-CGTGTTCGCGTTCAACTATG-3'
*BCAM2195 *rev	5'-ATGTTCCACGCCTTCTTCAC-3'
*BCAM2453 *for	5'-GATCCCGTACGTGAACGACT-3'
*BCAM2453 *rev	5'-ATAGACGATCTTGCCGTTCG-3'

### Assays for resistance to antimicrobial agents

To assess the resistance of strains to polymyxin B on solid media, cells were grown overnight in LB, diluted to an optical density at 600 nm (OD_600_) of 1.0 and serially diluted in ten-fold increments to 10^-4^. Ten-μl drops were spotted on to LB-agar plates supplemented with the vehicle control, 25 μg/ml, 200 μg/ml, or 1000 μg/ml polymyxin B. Plates were incubated for 24 h and then scanned. To assess the stability of the increased resistance to polymyxin B, experiments were carried out as described above using cells that had been grown in liquid culture for 120 h in the absence of polymyxin B (with passaging of cells to fresh media every 24 h).

To assess the resistance of the strains to polymyxin B in liquid media, bacteria were grown overnight and diluted to an OD_600 _of 0.01. Three hundred-μl volumes were aliquoted in 1.5 mL Eppendorf tubes, polymyxin B was added at final concentrations of 0 (vehicle control), 25, 50, 100, 200, 400, 600, 800, and 1000 μg/ml, cells were grown for 24 h while rotating in a LabQuake (Barnstead Thermolyne, Dubuque, Iowa) and the final OD_600 _was measured. Similar experiments were performed with the antimicrobial peptide honey bee melittin, except Mueller-Hinton Broth (MHB) was used instead of LB and final melittin concentrations used were 0 (vehicle control), 5, 25, 50, 100, and 200 μg/ml.

Resistance to SDS, novobiocin, gentamicin, tetracycline, chloramphenicol, and imipenem was assessed using disk diffusion assays. Briefly cells were grown overnight, diluted to an OD_600 _of 0.2 and spread onto LB-agar plates. Blank paper disks were added to plates and inoculated with 8 μl of 10% (wt/vol) SDS, 0.5% (wt/vol) novobiocin, 10% (wt/vol) gentamicin, 0.5% (wt/vol) tetracycline, 0.5% (wt/vol) chloramphenicol, or 0.5% (wt/vol) imipenem. Plates were incubated for 24 h and zones of inhibition were measured.

### Growth curves

Growth curves were completed as previously described for *B. cenocepacia *heptoseless mutants [14].

### Dansyl-polymyxin B binding and 1-N-phenylnaphthylamine (NPN) permeability assays

Dansyl-polymyxin B was synthesized and quantified using the dinitrophenylation assay as previously described [51,52]. Binding of dansyl-polymyxin B to whole *B. cenocepacia *cells was conducted as described by Ortega *et al *[15], cells were excited at 340 nm and emission at 485 nm was measured. Assays for NPN permeability were conducted similarly to the dansyl-polymyxin B assay except cells were treated with 20 μl of 50 μM of NPN in acetone. Cells were then excited at 350 nm and emission at 420 nm was measured.

### Measurement of adherent growth in polymxyin B

Experiments were conducted as described above for the polymyxin B liquid challenge. The OD_600 _was recorded from cultured cells prior to vortexing tubes and after vigorous vortexing of tubes. A ratio of OD_600 _after vortexing to OD_600 _before vortexing was calculated.

### Microscopic assessment of colony morphology

Cells were plated for isolated colonies on LB-agar plates and incubated for 48 h. Changes in colony morphology were recorded at 100× magnification using an Olympus IX71 inverted microscope and Image-Pro Plus Version 5.0 software.

### Growth of bacteria and RNA extraction for microarray analysis

Bacteria were grown overnight and then diluted to an optical density at 600 nm (OD_600_) of 0.05 in 30 ml of LB. Cells were grown for 3 h to an OD_600 _between 0.3 and 0.4 and then treated with either polymyxin B (500 μg/ml for K56-2 and RSF34 4000B, 25 μg/ml for RSF34) or the vehicle control for 30 min (final OD_600 _between 0.4 and 0.5). RNA was prepared from *B. cenocepacia *using the RiboPure-Bacteria kit from Ambion, Inc. (Austin, TX, USA) and treated with DNAse 1 (also from Ambion), following the manufacturer's protocol; each treatment condition required RNA prepared with five columns of the kit. RNA from the five individual preparations were combined and concentrated with LiCl. Integrity of the RNA was assessed by agarose gel electrophoresis and by measuring the ratio of absorbance at 260 nm to 280 nm (values obtained between 2.0 and 2.2). RNA was subjected to a PCR reaction for the gene *hisD *using Taq Polymerase (Qiagen Inc., Mississauga, ON, Canada), primers *hisD *for and *hisD *rev (Table 4), and the following reaction conditions: 94°C for 2 min, 29 cycles of 94°C for 40 sec, 60°C for 40 sec, and 72°C for 40 sec, followed by a final extension at 72°C for 7 min. If a reaction product was PCR amplified the RNA sample was treated with DNAse 1 again and re-tested. RNA was prepared in three independent experiments for microarray analysis.

### Microarray analysis

Labeled cDNA was synthesized from RNA and hybridized as previously described [48,53] to a custom 2 × 11 K *B. cenocepacia *microarray developed with Agilent's two-color 60-mer inkjet synthesis platform (Agilent Technologies, Santa Clara, CA, USA). For each of the five comparisons (Additional file 6 Figure S5), the two sample cDNA pools were fluorescently labeled with either Cy3 or Cy5 dyes and directly compared to each other. For each of these comparisons, one of the biological replicate experiments was re-analyzed with the dyes swapped.

Data was imported into GeneSpring (version 7.3.1) and normalized using the "Agilent FE" procedure. Genes listed in the additional files were found to be significantly different between the two test conditions (t-test p-value < 0.05), passed the Benjamini-Hochberg false discovery rate test, and were differentially regulated by 2-fold or more (as an arbitrarily chosen cut-off).

The microarray dataset has been deposited in the ArrayExpress database http://www.ebi.ac.uk/arrayexpress/ under accession number E-MTAB-720.

### Real-time PCR

Primers used for real-time PCR are listed in Table 4. All primer pairs had PCR efficiencies greater than 88%. RNA was extracted from 5 mL cultures as described above for bacterial growth and RNA extraction for microarray analysis. RNA was converted to cDNA using Transcriptor Reverse Transcriptase (Roche Diagnostics, Laval, Quebec, Canada) according to the manufacturer's instructions with modifications previously described [16]. Real-time PCR reactions using FastStart SYBR Green Master (Roche Diagnostics) and a Rotor-Gene 6000 thermal cycler (Corbett Life Sciences, Sydney, Australia) were conducted as previously described [16]. Data was analyzed using the manufacturer's software (Rotor-Gene 6000 Series Software Version 1.7). Fidelity of PCR amplifications was assessed using melt curve analysis and agarose gel electrophoresis. Fold changes in gene expression were calculated using the Pfaffl Method [54]. All changes are relative to an internal control, *hisD*, a gene which previously used as an internal control for semi-quantitative and real-time PCR analysis [16,55] and that was not found to be differentially expressed in any of the microarray analysis generated in this study.

### Motility assays

For swimming motility, bacteria were grown overnight in liquid culture and diluted to an OD_600 _of 1.0 in LB. Two-μl of culture was added to semi-solid LB plates (solidified with 0.3% Bacto Agar) by puncturing the top of the agar. Plates were incubated lid side up for 24 h and growth was measured as the diameter across which the bacteria grew. Swarming motility assays were completed as above except that media consisted of Nutrient Broth + 0.2% Glucose solidified with 0.5% Bacto Agar and bacteria were spotted on to the surface of the plates.

### Statistical analyses

All other statistical analyses (unpaired student's t-tests) were conducted with GraphPad Prism 4.0.

## Authors' contributions

SAL conceived the project, designed the experiments, prepared RNA for microarray analysis, conducted most experiments, analyzed data, wrote the manuscript, and edited the manuscript with the other authors. CC conducted experiments, analyzed data, and edited the manuscript. FDL and AM conducted sugar and fatty acid analyses, analyzed MS data, and wrote relevant sections of the manuscript. MAV conceived the project, obtained funding for the project, designed the experiments, analyzed data, and edited the manuscript. All authors read and approved the final manuscript.

## Supplementary Material

Additional file 1 **Figure S1 - LPS patterns and growth are maintained in each of the polymyxin B-resistant RSF34 isolates**. Click here for file

Additional file 2 **Figure S2 - Negative ion MALDI mass spectrum of the mutant RSF34 4000B LPS**. Click here for file

Additional file 3 **Figure S3 - Polymxyin B resistance is stable for at least 120 hr**. Click here for file

Additional file 4 **Figure S4 - Colony morphology varies in polymyxin B-resistant isolates**. Click here for file

Additional file 5 **Table S1. Genes up- and downregulated in RSF34 and RSF34 4000B in the presence of the vehicle control**. Sheet 1 lists genes transcriptionally upregulated (top) and downregulated (bottom) by *B. cenocepacia *strain RSF34 compared to K56-2 in the vehicle control. Sheet 2 lists genes transcriptionally upregulated (top) and downregulated (bottom) by *B. cenocepacia *strain RSF34 4000B compared to RSF34 in the vehicle control. Click here for file

Additional file 6 **Figure S5 - RSF34 and RSF34 4000B have significant defects in swimming and swarming motility**. Click here for file

Additional file 7 **Table S2. Genes up- and downregulated in K56-2, RSF34, and RSF34 4000B in the presence of polymyxin B**. Sheet 1 shows Genes transcriptionally upregulated (top) and downregulated (bottom) by *B. cenocepacia *strain K56-2 in the presence of polymyxin. Sheet 2 shows Genes transcriptionally upregulated (top) and downregulated (bottom) by *B. cenocepacia *strain RSF34 in the presence of polymyxin B. Sheet 3 shows Genes transcriptionally upregulated (top) and downregulated (bottom) by *B. cenocepacia *strain RSF34 4000B in the presence of polymyxin B. Click here for file
