# Supplementary material for: Transcriptional responses of Burkholderia cenocepacia to polymyxin B in isogenic strains with diverse polymyxin B resistance phenotypes
Source: BMC Genomics. 2011 Sep 29;12:472. doi: 10.1186/1471-2164-12-472 (PMC3190405; doi:10.1186/1471-2164-12-472)
Supplement: Additional file 1 — Figure S1 - LPS patterns and growth are maintained in each of the polymyxin B-resistant RSF34 isolates. [file 1471-2164-12-472-S1.DOC]

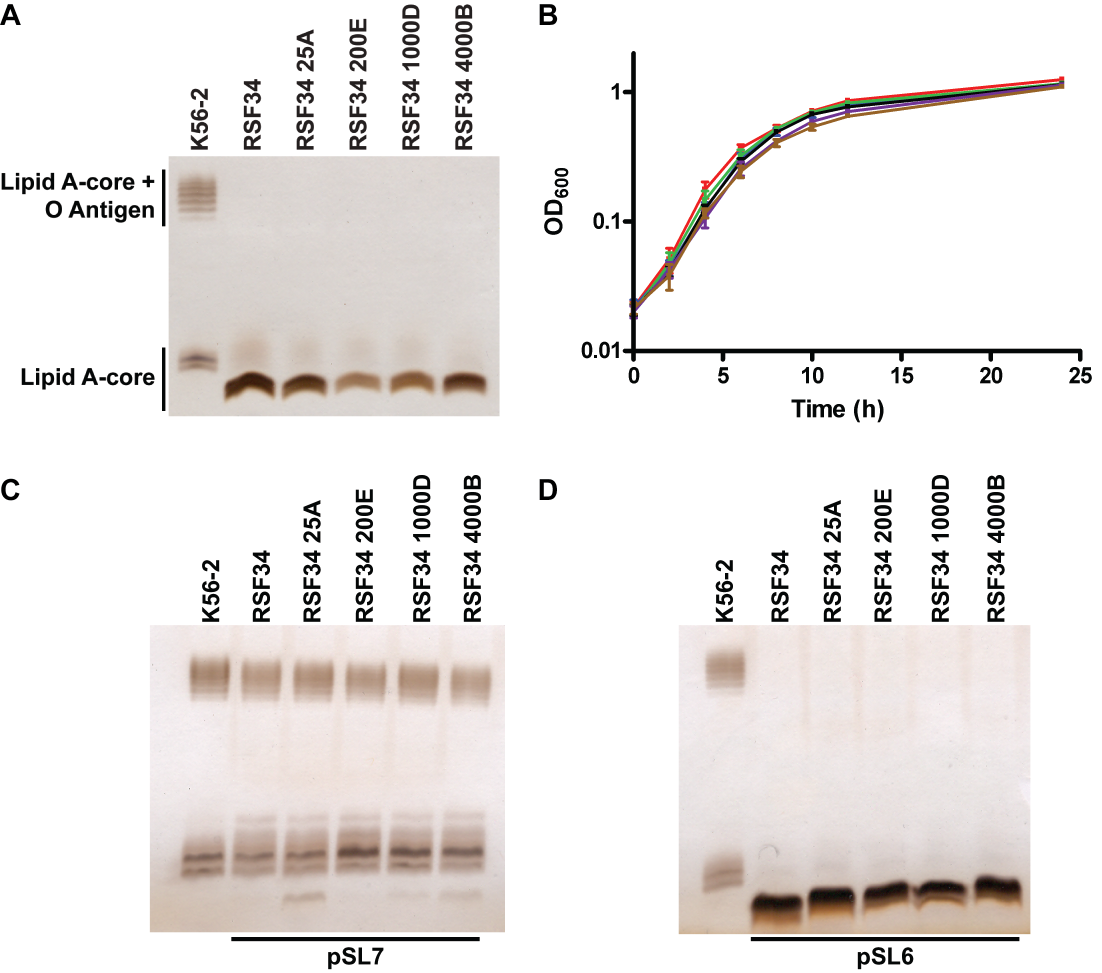


**Additional file 1: Figure S1 -** **LPS patterns and growth are maintained in each of the polymyxin B-resistant RSF34 isolates.** No changes were detected in the LPS profiles of mutants without (A) or with the cloning vector pSL6 (D). Introduction of the *hldA and hldD* genes encoded by pSL7 (C) results in the restoration of the parental LPS phenotype. No changes were noted in growth under standard laboratory conditions (B) for any of the polymyxin B-resistant RSF34 isolates compared to RSF34. Data in (B) show the means and standard error of the means for three experiments for K56-2 (red), RSF34 (blue), RSF34 25A (green), RSF34 200E (black), RSF34 1000D (purple), RSF34 4000B (brown).
