# Supplementary material for: Transcriptional responses of Burkholderia cenocepacia to polymyxin B in isogenic strains with diverse polymyxin B resistance phenotypes
Source: BMC Genomics. 2011 Sep 29;12:472. doi: 10.1186/1471-2164-12-472 (PMC3190405; doi:10.1186/1471-2164-12-472)
Supplement: Additional file 2 — Figure S2 - Negative ion MALDI mass spectrum of the mutant RSF34 4000B LPS. [file 1471-2164-12-472-S2.DOC]

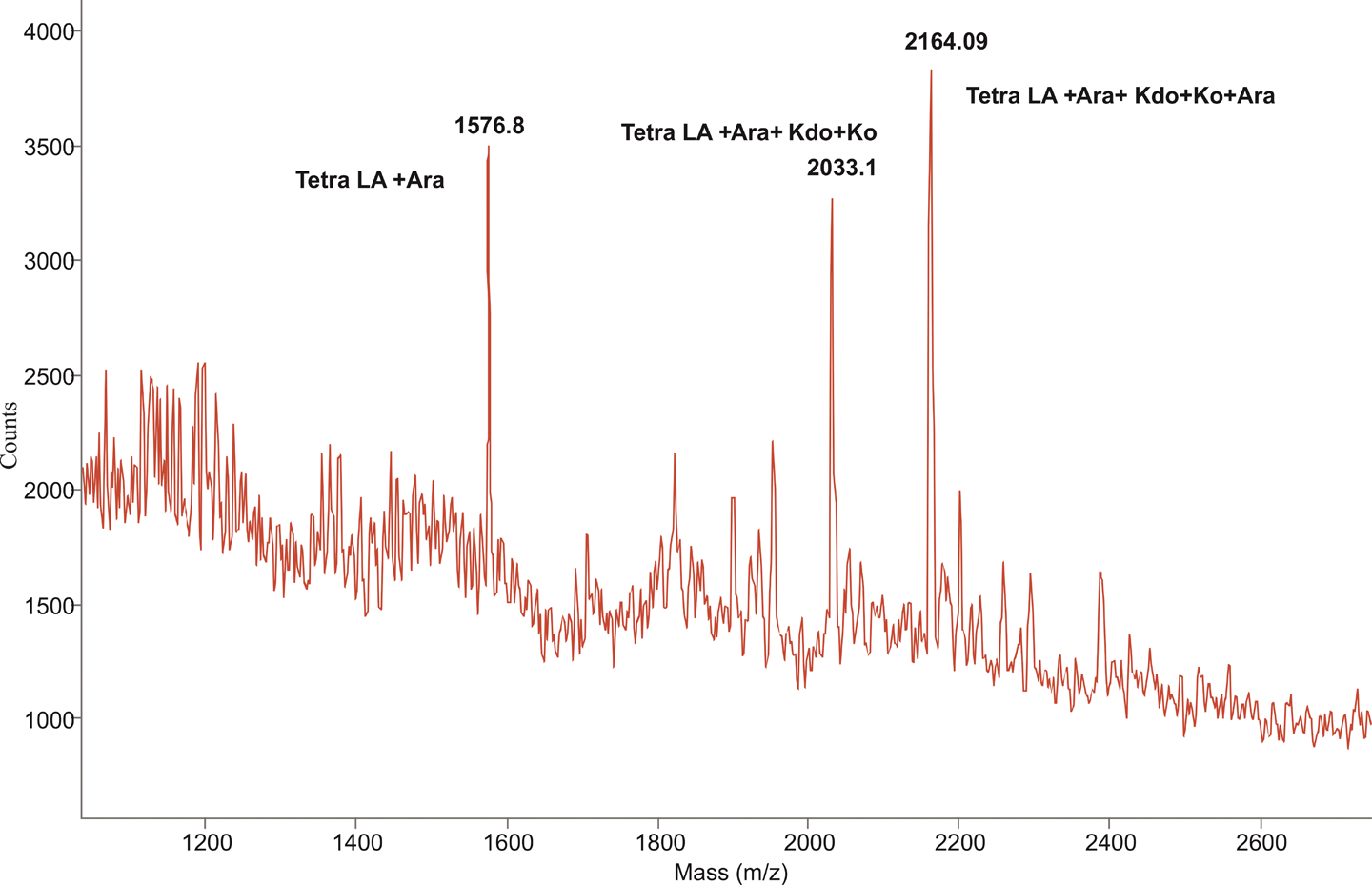


**Additional file 2 Figure S2 -** **Negative ion MALDI mass spectrum of the mutant RSF34 4000B LPS**. MALDI MS analysis of intact sample showed the presence of a few ion peaks in the lipid A low molecular weight region and also some other peaks in the region around 2300 Da. The major LPS lipid A species was composed of a tetra-acylated lipid A lacking a 14:0(3OH) and bearing stoichiometric 4-amino-4-deoxy-L-arabinose (l-Ara4N) on the lipid A. In the inner core region, contained the disaccharide Kdo-Ko and a second l-Ara4N residue. Only a few minor peaks accounted for a third l-Ara4N. Compositional analysis by acetylated *O*-methyl glycoside derivatives revealed the presence of glucosamine (GlcN), 3-deoxy-D-*manno*-oct-2-ulopyranosonic acid (D-Kdo), D-*glycero*-D-*talo*-oct-2-ulopyranosonic acid (D-Ko), and L-Ara4N; no traces of heptose and hexose residues were found, as expected given the lack of a heptose biosynthesis gene in this mutant. Fatty acid analysis revealed (*R*)-3-hydroxyhexadecanoic (16:0(3OH)) in amide-linkage and (*R*)-3-hydroxytetradecanoic (14:0(3OH)) and tetradecanoic acid (14:0) in ester-linkage. LA, lipid A.
