# Supplementary material for: Transcriptional responses of Burkholderia cenocepacia to polymyxin B in isogenic strains with diverse polymyxin B resistance phenotypes
Source: BMC Genomics. 2011 Sep 29;12:472. doi: 10.1186/1471-2164-12-472 (PMC3190405; doi:10.1186/1471-2164-12-472)
Supplement: Additional file 3 — Figure S3 - Polymxyin B resistance is stable for at least 120 hr. [file 1471-2164-12-472-S3.DOC]

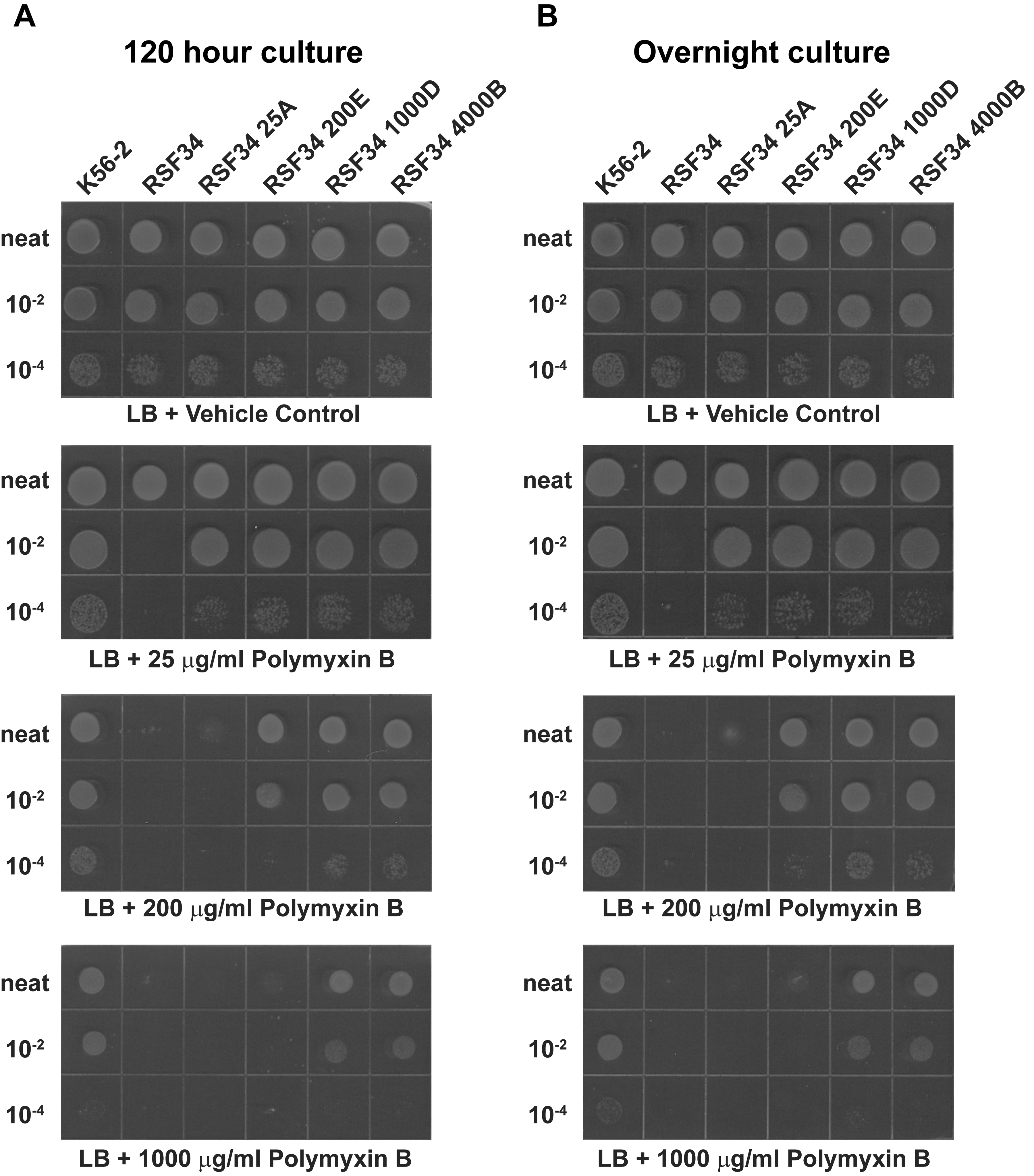


**Additional file 3 Figure S3 - Polymyxin B resistance is stable for at least 120 hr.** Cells were grown for either 120 hours (A) or overnight (B) in the absence of selection and then plated and challenged with polymyxin B. Shown is a representative image from two independent experiments, neat is equal to an OD_600_ of 1.0.
