# Supplementary material for: Transcriptional responses of Burkholderia cenocepacia to polymyxin B in isogenic strains with diverse polymyxin B resistance phenotypes
Source: BMC Genomics. 2011 Sep 29;12:472. doi: 10.1186/1471-2164-12-472 (PMC3190405; doi:10.1186/1471-2164-12-472)
Supplement: Additional file 4 — Figure S4 - Colony morphology varies in polymyxin B-resistant isolates. [file 1471-2164-12-472-S4.DOC]

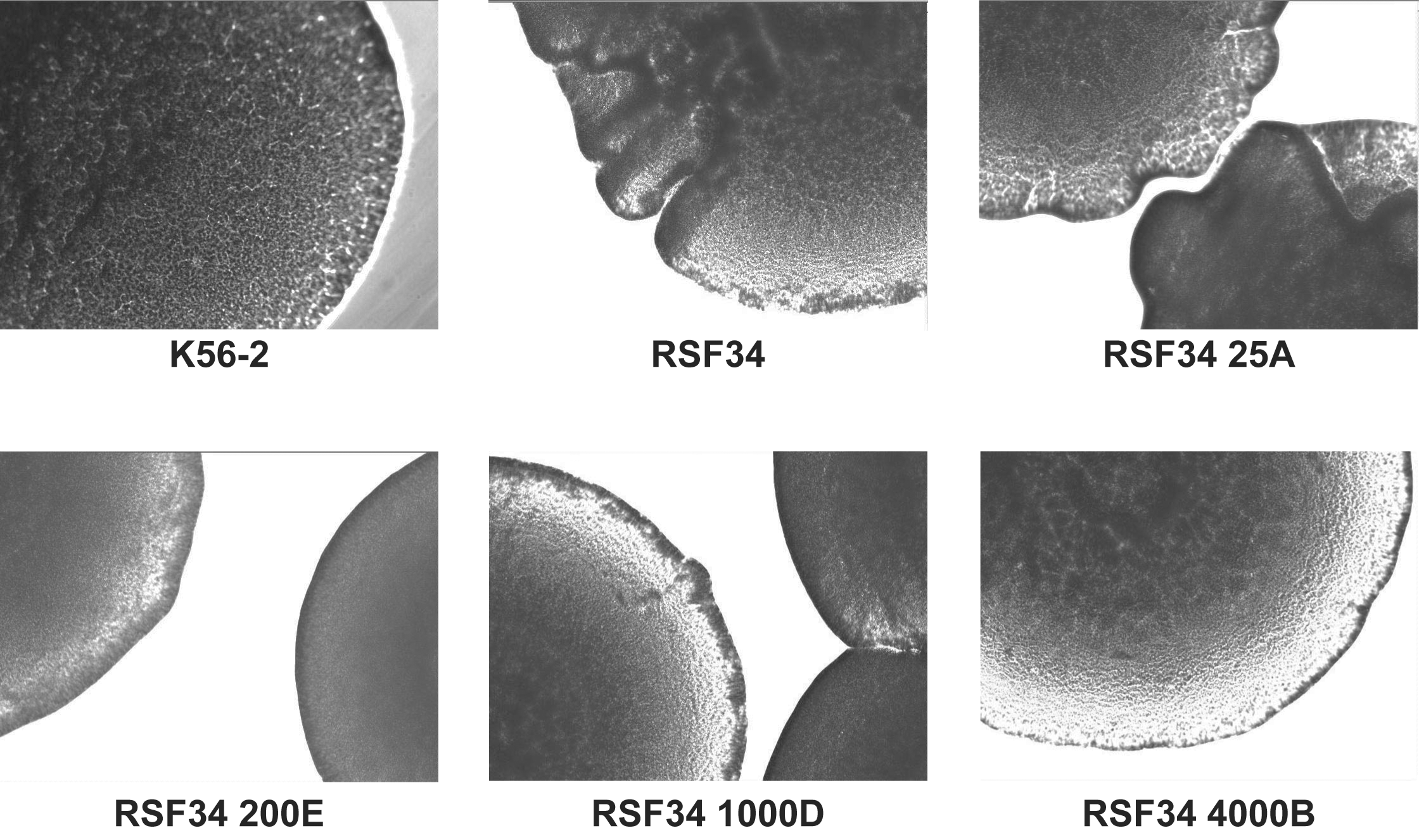


**Additional file 4 Figure S4 - Colony morphology varies in polymyxin B-resistant isolates.** Images representative of multiple colonies from three independent experiments were obtained with an Olympus IX71 inverted microscope at 100X magnification.
