# Supplementary material for: Transcriptional responses of Burkholderia cenocepacia to polymyxin B in isogenic strains with diverse polymyxin B resistance phenotypes
Source: BMC Genomics. 2011 Sep 29;12:472. doi: 10.1186/1471-2164-12-472 (PMC3190405; doi:10.1186/1471-2164-12-472)
Supplement: Additional file 6 — Figure S5 - RSF34 and RSF34 4000B have significant defects in swimming and swarming motility. [file 1471-2164-12-472-S6.DOC]

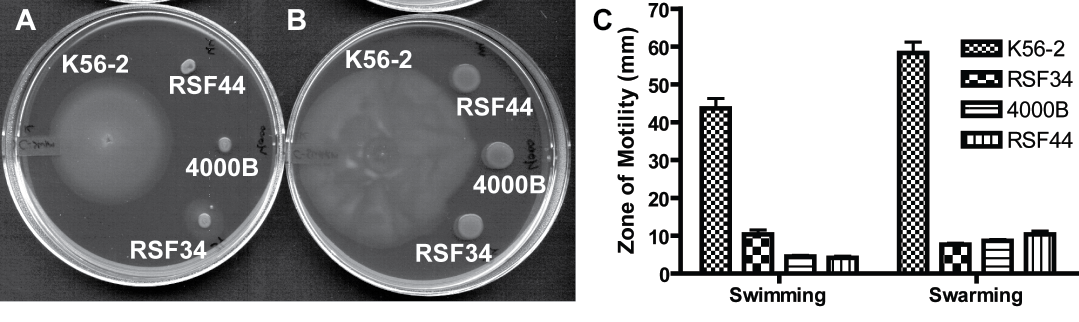


**Additional file 6 Figure S5 - RSF34 and RSF34 4000B have significant defects in swimming and swarming motility.** Bacteria were inoculated into swimming motility plates (A) or on swarming motility plates (B). Images shown are representatives from three independent experiments. Diameters across the zones of bacterial swimming or swarming were measured and the means and standard errors of the means for the three independent experiments are shown in (C).
